# Changes in the burden of malaria following scale up of malaria control interventions in Mutasa District, Zimbabwe

**DOI:** 10.1186/1475-2875-12-223

**Published:** 2013-07-01

**Authors:** Sungano Mharakurwa, Susan L Mutambu, Joseph Mberikunashe, Philip E Thuma, William J Moss, Peter R Mason

**Affiliations:** 1 Johns Hopkins Malaria Research Institute, Bloomberg School of Public Health, Johns Hopkins University, 615 North Wolfe Street, Baltimore, MD 21205, USA; 2 Macha Research Trust, Namwala Road, P.O. Box 630166, Choma, Zambia; 3 National Institute of Health Research, P.O. Box 573 Harare, Zimbabwe; 4 National Malaria Control Programme, Ministry of Health and Child Welfare, Harare, Zimbabwe; 5 Biomedical Research and Training Institute, Nicoz Diamond House, Samora Machel Ave, P.O. Box CY1753, Harare, Zimbabwe; 6 College of Health Sciences University of Zimbabwe, P.O. box A 178, Avondale Harare, Zimbabwe

**Keywords:** Malaria, Epidemiology, Transmission, Control, Prevalence, Zimbabwe

## Abstract

**Background:**

To better understand trends in the burden of malaria and their temporal relationship to control activities, a survey was conducted to assess reported cases of malaria and malaria control activities in Mutasa District, Zimbabwe.

**Methods:**

Data on reported malaria cases were abstracted from available records at all three district hospitals, three rural hospitals and 25 rural health clinics in Mutasa District from 2003 to 2011.

**Results:**

Malaria control interventions were scaled up through the support of the Roll Back Malaria Partnership, the Global Fund to Fight AIDS, Tuberculosis and Malaria, and The President’s Malaria Initiative. The recommended first-line treatment regimen changed from chloroquine or a combination of chloroquine plus sulphadoxine/pyrimethamine to artemisinin-based combination therapy, the latter adopted by 70%, 95% and 100% of health clinics by 2008, 2009 and 2010, respectively. Diagnostic capacity improved, with rapid diagnostic tests (RDTs) available in all health clinics by 2008. Vector control consisted of indoor residual spraying and distribution of long-lasting insecticidal nets. The number of reported malaria cases initially increased from levels in 2003 to a peak in 2008 but then declined 39% from 2008 to 2010. The proportion of suspected cases of malaria in older children and adults remained high, ranging from 75% to 80%. From 2008 to 2010, the number of RDT positive cases of malaria decreased 35% but the decrease was greater for children younger than five years of age (60%) compared to older children and adults (26%).

**Conclusions:**

The burden of malaria in Mutasa District decreased following the scale up of malaria control interventions. However, the persistent high number of cases in older children and adults highlights the need for strategies to identify locally effective control measures that target all age groups.

## Background

Reductions in the burden of malaria have been reported throughout sub-Saharan Africa following the scale up of interventions under the Roll Back Malaria Partnership, the Global Fund to Fight AIDS, Tuberculosis and Malaria, The President’s Malaria Initiative and other public-private partnerships [1]. While these interventions contributed to the decline in malaria burden in many countries, the impact has not been universal, both within and between countries [1,2]. Within southern Africa, wide heterogeneity exists in the burden of malaria following the scale up of malaria control measures. Understanding why there has been sustained malaria reduction in some areas while in others these interventions have not had the same impact is critical to extending locally adapted malaria control efforts, developing new control strategies and achieving malaria elimination in southern Africa.

Reductions in malaria incidence in some cases pre-dated the introduction of widespread intervention efforts [1,3], suggesting that other as yet uncharacterized factors are responsible for the reduction in malaria transmission. Multidisciplinary research to ascertain what such factors may be and their relative contribution to the declining burden of malaria is imperative to ensure the effectiveness and sustainability of malaria control and elimination efforts.

The International Centre of Excellence for Malaria Research (ICEMR) in southern Africa aims to better understand the malaria epidemiology, vector biology and parasite genomics in three contrasting epidemiological settings of malaria transmission in Zambia and Zimbabwe [4,5]. A survey was conducted of reported malaria cases and control interventions in Mutasa District, eastern Zimbabwe to better understand trends in the burden of malaria and their temporal relationship to control activities.

## Methods

### Study area and population

Mutasa District has a population of approximately 180,000 persons and is located in a mountainous area of Manicaland Province in eastern Zimbabwe bordering Mozambique. The altitude ranges from less than 600 to more than 1,600 m above sea level. The low-lying regions of the district, called the Honde Valley, are endemic for malaria and encompass the valleys of the Honde and Pungwe rivers.

Honde Valley consists largely of farmland and several large tea estates. Workers on the estates live in compounds consisting of small housing communities but much of the area consists of small-scale subsistence farms characterized by small villages. In general, the village settlements are concentrated along perennial streams and on hillsides endowed with arable soils. While the streams emanating from the surrounding mountains flow throughout the year, Honde Valley experiences cool (May to July) and hot (August to October) dry periods, with a rainy season extending from November to April. Malaria transmission is seasonal, unstable and epidemic, exacting morbidity and mortality across all ages [6-9]. The vector mosquito species responsible for malaria transmission are not well characterized, although data from the Zimbabwe National Institute of Health Research (NIHR) insecticide resistance surveillance suggest that *Anopheles gambiae* s.s. is the major malaria vector (Lukwa Nzira 2012, personal communication).

### Data collection

**Figure 1:**
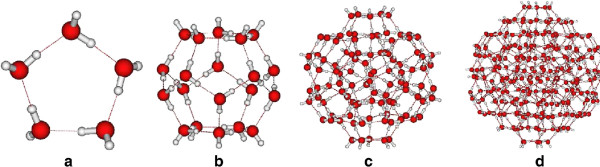
Mutasa District map and location of health care facilities.

In Mutasa District, health care is delivered through government, missionary and estate health centres, with three district hospitals (Bonda Mission, Hauna and Old Mutare), six rural hospitals and 37 rural health clinics. Data on staffing, vector control interventions, the number of clinical and confirmed cases of malaria and treatment regimens from 2003 to 2011 were collected using a standardized data collection instrument from all three district hospitals and twenty-eight representative health care centers selected by simple random procedure, which included three of the six rural hospitals and 25 of the 37 clinics (Figure 1). Complete data were not available for all clinics as some were established during the study period, and data for 2011 spanned only the first quarter, coinciding with part of the peak malaria transmission season (February to May). Information on coverage with indoor residual insecticide spraying (IRS) and insecticide-treated bed nets (ITNs) was obtained from the NIHR and the National Malaria Control Programme of the Ministry of Health & Child Welfare that is responsible for implementation of malaria control interventions.

## Results

### Health facility staffing

Only the one district and two mission hospitals had a full-time physician or clinical officer on staff, while the rural hospitals and one estate clinic had physicians who visited weekly. The remaining facilities relied on nursing staff, with state-registered nurses (SRNs) or state-certified nurses (SCNs), primary care nurses (PCNs) and nurse aides (NAs) available to seven, 23 and 30 of the 31 health care facilities, respectively. SRNs undergo three-year general nurses’ professional diploma training, SCNs receive a two-year professional training diploma, and PCNs receive one-year basic training. The district and mission hospitals were the only facilities with laboratory staff capable of performing microscopy. A pharmacy technician was employed only at Bonda Mission Hospital and Hauna Hospital. However, all but seven health care facilities had at least one environmental health technician (EHT) responsible for environmental and disease surveillance.

### Vector control activities

Zimbabwe has had a national malaria control programme comprising vector control with IRS, distribution of ITNs and case management for more than 60 years [10]. Despite economic constraints from 2003 to 2011, vector control continued in Mutasa District. Mutasa District was one of the first districts to be included in the Roll Back Malaria Programme since its launch in Zimbabwe in 2001 and subsequent scale up for impact [10]. Both IRS and ITNs were used for vector control. Although data on coverage with ITNs were not available, the estimated proportion of the population protected by IRS in Mutasa District was 92% and 93% in 2010 and 2011 [11].

### Malaria diagnostics

Before 2006, most cases of malaria were treated based on clinical criteria, as the availability of microscopy was limited to hospitals. With the introduction of RDTs, access to accurate diagnosis was scaled up, such that all health facilities had access to RDT by 2008. From 2008 to 2011, during which time more than two thirds of suspected cases were tested by RDT, 64% to 77% of suspected malaria cases were RDT positive (Table 1).

**Table 1:** Reported malaria cases, diagnostic tests and treatment regimens by year at health care facilities in Mutasa District, 2003 – 2011

**Year**		**2003**	**2004**	**2005**	**2006**	**2007**	**2008**	**2009**	**2010**	2011*
**Number of reporting health care facilities**		24	27	28	29	31	31	31	31	31
**Reported number of suspected malaria cases**	Total	31,782	50,630	62,933	79,994	74,226	101,531	73,619	61,607	50,798
	< 5 years	7,974	12,464	13,948	15,611	15,812	22,978	14,991	12,035	12,476
	≥ 5 years	23,808	38,166	48,985	64,383	58,414	78,553	58,628	49,572	38,322
**Proportion of suspected malaria cases by age category**	< 5 years	25%	25%	22%	20%	21%	23%	20%	20%	25%
	≥ 5 years	75%	75%	78%	80%	79%	77%	80%	80%	75%
**Reported number of diagnostic tests performed**	Microscopy	324	187	796	850	22	9	46	0	0
	RDT	0	0	704	702	11996	65868	62912	50717	39207
**Proportion of suspected malaria cases tested by RTD**		0%	0%	1%	1%	16%	65%	85%	82%	77%
**Reported number of RDT positive malaria cases**	Total	0	0	328	322	6561	50462	44326	32641	28098
	< 5 years	0	0	124	50	1098	13788	8565	5471	5631
	≥ 5 years	0	0	204	272	5463	36674	35761	27170	22467
**Proportion of suspected malaria cases confirmed by RDT**	Total	0%	0%	47%	46%	55%	77%	70%	64%	72%
	< 5 years	0%	0%	41%	58%	53%	78%	73%	58%	57%
	≥ 5 years	0%	0%	51%	44%	55%	76%	70%	66%	77%
**Reported number of treated cases by drug regimen**	Total	31,782	51,630	62,933	79,994	74,226	101,531	73,619	61,607	50,798
	CQ	20,151	15,838	18,886	20,129	19,320	13,924	0	0	0
	CQ + S/P	11,631	35,792	44,047	59,865	41,764	2,955	344	0	0
	ACT	0	0	0	0	13,142	84,652	73,275	61,607	50,798
**Reported number of non-malaria cases**	Total	114,148	143,130	171,116	163,153	171,630	162,524	177,578	184,396	96,826
	< 5 years	24,962	30,791	36,919	29,773	28,170	46,325	58,464	65,515	34,501
	≥ 5 years	89,186	112,339	134,197	133,380	143,460	116,199	119,114	118,881	62,325
**Proportion of suspected malaria cases of all clinic cases**	< 5 years	24 %	29%	27%	34%	36%	33%	20%	16%	27%
	≥ 5 years	21%	25%	27%	33%	29%	40%	33%	29%	38%

* First quarter only.

### Malaria treatment regimens

**Figure 2:**
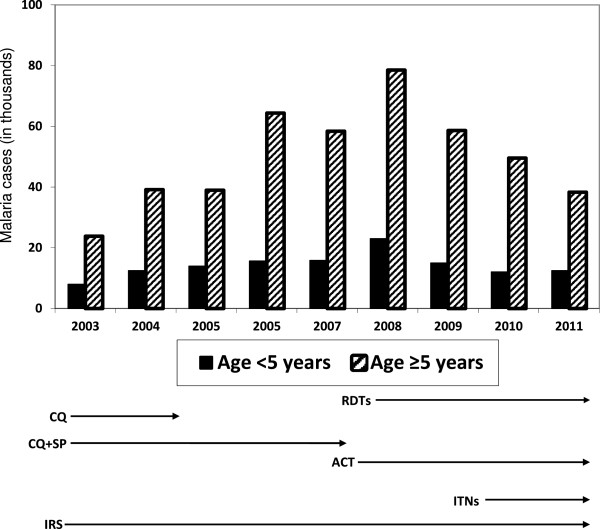
Number of suspected malaria cases by year in persons younger and older than five years of age in Mutasa District.

Anti-malarial treatment regimens changed from chloroquine and chloroquine plus sulphadoxine-pyrimethamine to artemisinin combination therapy (ACT) in 2007 (Figure 2). Chloroquine alone was no longer used after 2008 and ACT was the sole treatment regimen after 2009 (Table 1).

### Changing burden of malaria

The number of suspected cases of malaria increased from 2003 to a peak in 2008 but then declined 39% from 2008 to 2010 (Figure 2). The proportion of all suspected cases of malaria in children younger than five years of age ranged from 20 to 25%. Conversely, the proportion of all suspected cases of malaria in older children and adults remained high from 2003 to 2011, ranging from 75 to 80%. From 2008 to 2010, the overall number of RDT positive cases of malaria decreased 35% but the decline was greater for children younger than five years of age (60%) compared to older children and adults (26%).

## Discussion

This malaria survey in Mutasa District showed a decline in suspected number of malaria cases from a peak in 2008 following increased coverage with malaria control interventions. What was striking was the greater decline in RDT-positive cases among children younger than five years compared to older children and adults. As scale up of diagnostics, effective treatment regimens and vector control was implemented, the asymmetric decrease in malaria among different age groups poses a challenge to control and elimination efforts.

Although the malaria vectors in Mutasa District are not well characterized, malaria transmission increased despite coverage with IRS and ITNs [12,13]. Coverage with vector control interventions as reported by the National Malaria Control Programme, particularly for ITNs, was higher in Mutasa District than that reported for many endemic countries [14], although district level data do not reflect national or provincial coverage levels. The modest impact of these control methods, particularly in older children and adults, may indicate vector species that are biologically or behaviourally resistant to the interventions. Presumably, indoor-based interventions worked better in protecting young children but not persons of older ages who are more likely to be bitten during farming and other occupational activities.

The malarial burden in Africa remains heterogeneous among areas undergoing apparently similar intervention efforts [3]. In Ethiopia, there was a >60% reduction in malaria incidence following the introduction of IRS [15], but resurgent malaria despite extensive availability of ITNs was reported from Kenya [16]. Cryptic outdoor-feeding vectors have been reported recently from western Kenya as a potential cause of sustained transmission in areas with high coverage with IRS or ITNs [17]. Extensive use of ITNs has been reported to alter the composition of the vector complex, with relative reductions in *An. gambiae* s.s. and *Anopheles funestus* compared with *Anopheles arabiensis*[16,18]. Changes in the composition of the vector complex in Tanzania paralleled a dramatic decline in malaria transmission [19]. In areas where *An. gambiae* has acquired resistance to pyrethroids, ITNs are no more effective than untreated nets in reducing biting [20]. Clearly, understanding the vector biology in Mutasa District is critical to designing effective and sustainable control strategies.

Overdiagnosis of malaria based on clinical suspicion is a known problem in Africa [21] and elsewhere, and confirmatory diagnosis is recommended. RDTs have been shown to be a cost-effective measure to ensure malaria treatment is directed to those who are infected [22,23]. In Mutasa District, the use of RDT increased to approximately 80% of suspected cases and up to 75% of RDT were positive. The trend of increasing RDT positivity despite malaria control measures could reflect improved health staff selection of patients likely to have malaria. However, the improvement would have been specific for individuals older than five years. The proportion of all clinic cases that are RDT positive is known to decline when the burden of malaria is reduced, as other conditions become the primary cause for febrile presentations [24].

Modest reductions in the prevalence of malaria were observed following the introduction of ACT in Tanzania [25] and other areas in sub-Saharan Africa. These reductions in malaria prevalence appear to have occurred, in some areas, prior to local scale up of the vector control and the introduction of ACT. Low malaria prevalence hinders the ability to measure the impact of malaria control interventions. For example, an attempt to show the efficacy of gametocidal treatment in reducing malaria transmission in a low transmission region of Tanzania was not possible because of the reduction in malaria transmission in control villages [26].

## Conclusions

The burden of malaria in Mutasa District decreased following the scale up of malaria control interventions. However, the persistent high number of cases in older children and adults highlights the need for strategies to identify locally effective control measures that target all age groups.

## Competing interests

The authors declare that they have no competing interests.

## Authors’ contributions

SM worked on the design of the study, data analysis and write up of the manuscript. MM helped coordinate the field team, collected data and reviewed the manuscript. SLM helped in the design of the study, provided National Institute of Health Research data and reviewed the manuscript. JM was the director of the National Malaria Control Programme, provided health centre data and reviewed the manuscript. PET helped provide clinical expertise and contributed to analysis and write-up of the manuscript. WJM served as part of ICEMR programme directorate and provided guidance as well as review of the manuscript. PRM was the local country PI who initiated the survey, worked on the study design and contributed to data analysis and writing of the manuscript. All authors read and approved the final manuscript.
